# LYL1 Degradation by the Proteasome Is Directed by a N-Terminal PEST Rich Site in a Phosphorylation-Independent Manner

**DOI:** 10.1371/journal.pone.0012692

**Published:** 2010-09-10

**Authors:** Georgi L. Lukov, Margaret A. Goodell

**Affiliations:** 1 Department of Pediatrics, Baylor College of Medicine, Houston, Texas, United States of America; 2 Stem Cells and Regenerative Medicine Center, Baylor College of Medicine, Houston, Texas, United States of America; Roswell Park Cancer Institute, United States of America

## Abstract

**Background:**

The Lymphoblastic leukemia 1 (*LYL1*) gene is a proto-oncogenic transcription factor found upregulated in patients with T-cell acute lymphoblastic leukemia (T-cell ALL). Initially, the upregulation was described to be as a result of a translocation. However, further studies revealed that transcriptional upregulation of LYL1could also occur without translocations. In addition, post-translational mechanisms, such as protein degradation could influence LYL1 expression as well.

**Methodology/Principal Findings:**

In this study, we considered possible post-translational regulation of Lyl1, and investigated fundamental mechanisms governing LYL1 degradation in cell-based culture assays. We identify a PEST sequence motif located in the N-terminus of LYL1, which determines the efficiency of LYL1 degradation by the proteasome. The absence of the PEST degradation site leads to accumulation or upregulation of LYL1. We also show that LYL1 is phosphorylated by MAPK at S_36_, and determined that proteasomal degradation of LYL1 occurs in a phosphorylation-independent manner.

**Conclusions/Significance:**

Understanding LYL1 degradation is a step forward not only towards deciphering the normal function and regulation of LYL1, but could suggest post-translational mechanisms for upregulation of LYL1 that may contribute to its oncogenic role.

## Introduction

The lymphoblastic leukemia 1 (*LYL1*) gene codes for a basic helix-loop-helix (bHLH) transcription factor [1]. The basic region facilitates DNA interactions and the HLH domain, protein dimerizations [2], [3]. LYL1 has an important role in hematopoietic stem cell biology, normal hematopoiesis and leukemia. It is expressed throughout the hematopoietic lineages with the exception of T-cells [4], [5], [6]. Deletion of *Lyl1* in mice reduces the hematopoietic stem and progenitor populations and the mature B-cells [7]. Lyl1 is non-essential for embryonic development; however, deletion of *Lyl1* together with its paralog, the stem-cell leukemia (*Scl*) gene, causes rapid apoptosis of hematopoietic progenitors in adult mice [4]. Upregulation of LYL1 has been linked to a subtype of T-cell acute lymphoblastic leukemia defined by a stem-like phenotype and an unfavorable prognosis [8], [9]. In addition, significant proportion of the *Lyl1* transgenic mice develop T- and B-cell lymphoma after an average latent period of one year [10]. Furthermore, overexpression of Lyl1 in the mouse bone marrow causes hematopoietic progenitor-expansion and increased mature T-cells. These effects were most likely due to the anti-apoptotic and proliferative roles of the Lyl1 overexpression in the hematopoietic system [Lukov et al. – accepted for publication in Leukemia Research].

*LYL1* was first discovered ectopically expressed in T-cell ALL lymphoblasts as a result of the t(7;19)(q35;p13) translocation with the T-cell receptor beta chain gene [1], [11]. *LYL1* translocations, on average, have been observed in 2% of all T-cell ALL cases [12]. However, Ferrando at al. reported that 22% of the studied children with T-cell ALL have overexpressed LYL1 which was not associated with any locus-specific translocations of the *LYL1* gene [8]. In addition, a *LYL1* translocation and multiple translocation-independent upregulations have also been observed in acute myeloblastic leukemia (AML) cases [9], [13]. It is clear that there are multiple mechanisms responsible for LYL1 upregulation in ALL and AML [14]. What remains unknown is the nature of these mechanisms and how they contribute to the role of LYL1 in leukemia. Chan et al. reports that Ets and GATA factors regulate Lyl1 transcription however, very little is known about the protein stability and the post-translational regulation of LYL1 [15].

Disregulated protein degradation causing accumulation could be a powerful reason for increased gene expression [16], [17]. Post-translational processing and degradation are regulatory mechanisms controlling protein expression and function [18], [19]. Their role in the function of LYL1 is still unexplored. The aim of our study was to examine the mechanisms governing protein stability and degradation of LYL1. We determined whether LYL1 degradation is proteasome dependent. We identified and studied the importance of a PEST (Proline (P), Glutamate (E), Aspartate (D), Serine (S) and Threonine (T)) rich sequence motif, recognized as a site for protein degradation [20], [21]. In addition, we established that LYL1 is phosphorylated and further investigated how the phosphorylation affects LYL1 degradation. Our findings set a solid foundation for further basic science and clinical explorations aimed at understanding the mechanisms of LYL1 upregulation and their role in leukemia.

## Results

### LYL1 is degraded by the proteasome

**Figure 1:**
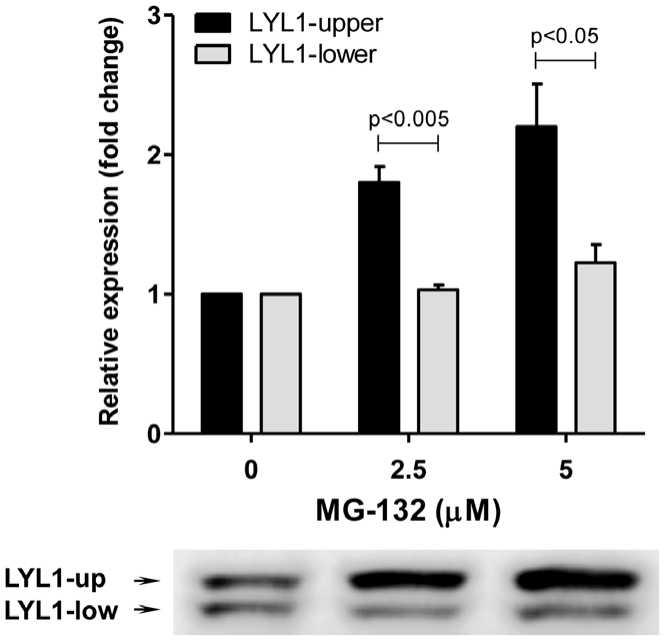
Accumulation of LYL1 as a result of proteasomal inhibition with MG-132. Cultured 293T cells, transiently transfected with wild type *LYL1*, were treated with 2.5 or 5 µM MG-132, or DMSO as a control, for 6 hrs under normal growth conditions. Following the incubation, the cells were lysed and LYL1 expression was analyzed by immunoblotting against the V5 tag. The bar graph represents the mean intensities of the upper and lower bands of LYL1 at increasing concentrations of MG-132 from four experiments. The error bars show the standard error of the mean (SEM). The p values were calculated using two tailed, unpaired t test.

For our studies we used the 293T cell-line which is a widely used and easily manipulated biological system shown to have the cellular machinery required for studies of ubiquitous processes, such as the protein proteasomal degradation [22], [23], [24]. The majority of the cellular proteins are degraded by the proteasome [25]. Therefore, our initial approach was to determine if LYL1 also is targeted for degradation by the proteasome. To test that, we treated cultured 293T cells, transfected with V5 tagged *LYL1* (LYL1-V5), with the proteasome inhibitor MG-132. We observed a dose dependent accumulation of LYL1 after 6 hrs of incubation with increasing concentrations of MG-132 (Fig. 1). Western blotting analysis revealed that the MG-132 induced accumulation affected predominantly the upper band of LYL1. Normally, on a western blot LYL1 is represented by two bands positioned approximately 5 kDa of each-other indicating, that there are two forms of LYL1. These forms could either be the wild type and a truncated form of LYL1 or most likely the wild type LYL1 existing in two post-translational modification states. If the latter is true, our observations would indicate that only the modified form of LYL1 is subjected to degradation with the modification itself acting as a signaling event.

### LYL1 is phosphorylated by the MAPK

**Figure 2:**
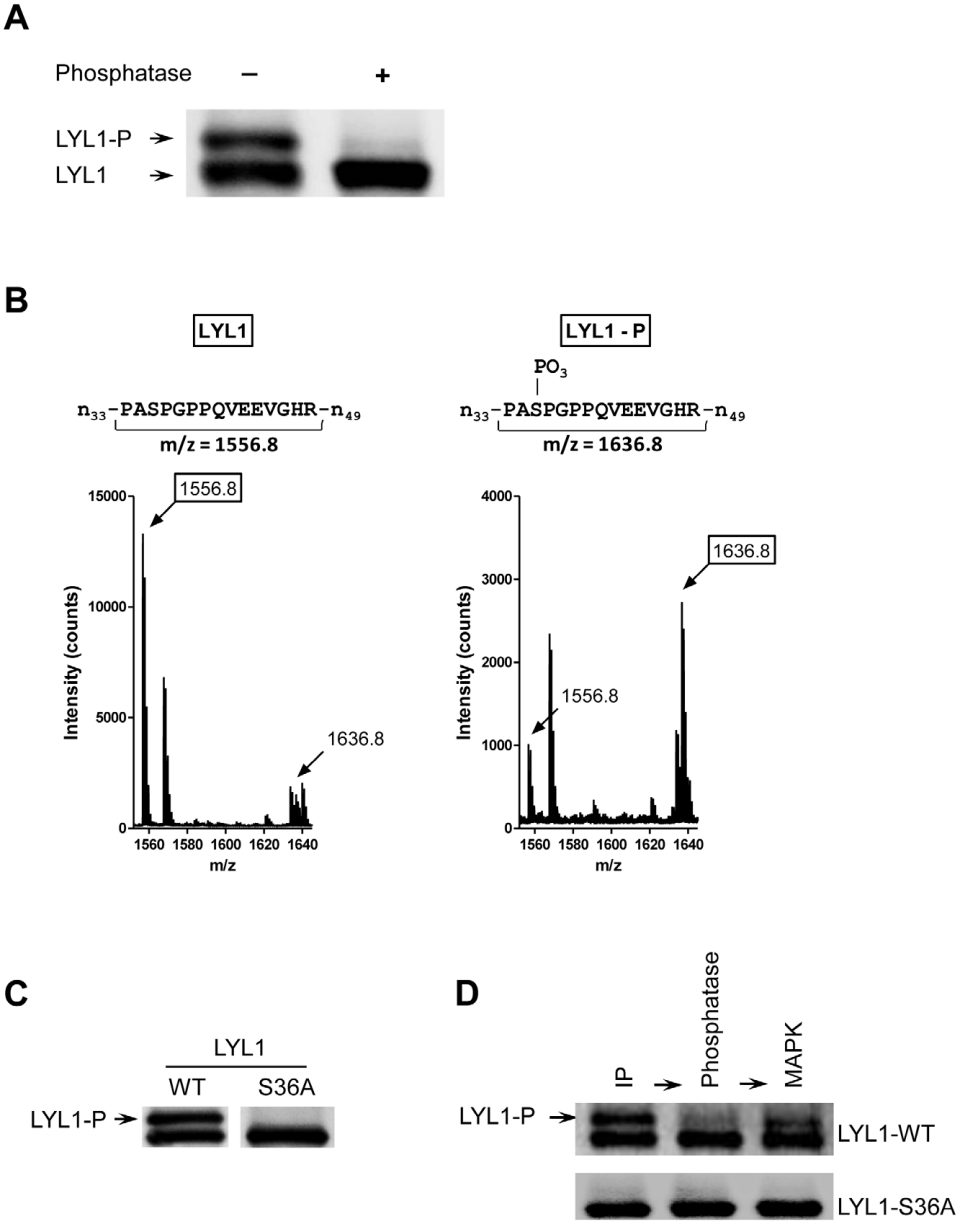
LYL1 is phosphorylated by MAPK at Serine 36. **A**) Dephosphorylation of LYL1 by Alkaline Phosphatase. Protein A/G beads with bound, immunoprecipitated LYL1-WT were treated with the Calf Intestinal Alkaline Phosphatase for 1 h. The samples were then analyzed by anti-V5 immunoblotting. The shown image is representative of four experiments. **B**) Mass fingerprinting of phosphorylated and non-phosphorylated LYL1. The upper, phosphorylated and the lower, non-phosphorylated bands of immunoprecipitated LYL1were excised, digested and mass fingerprinted by MALDI-TOF mass spectrometry. The histograms, representative of two experiments, show the measured m/z values of the peptide spanning residues 34 through 48 derived from the non-phosphorylated (LYL1) and the phosphorylated (LYL1-P) LYL1. **C**) Expression analysis of LYL1-WT and LYL1-S36A. LYL1 wild type and S36A variant were transiently expressed in 293T cells, immunoprecipitated and analyzed by western immunoblotting. **D**) Phosphorylation of LYL1 by the MAPK. Immunoprecipitated LYL1-WT and LYL1-S36 were first treated with Protein Phosphatase 1 to remove all phosphate modifications. Then, re-phosphorylation was attempted by treatment with the MAP kinase. The proteins were resolved and analyzed by anti-V5 immunoblotting. The images are representative of four experiments.

Common post-translational modifications are the protein phosphorylations. To confirm that LYL1 is a phosphorylated protein we immunoprecipitated LYL1 from 293T cells followed by treatment with Calf Intestinal Phosphatase (CIP) in order to remove any existing phosphate group modifications. Upon incubation with CIP the top band completely disappeared signifying that indeed LYL1 is phosphorylated and that the phosphorylated form has decreased electrophoretic mobility represented by the upper band (Fig. 2A).

Naturally, our next aim was to indentify and localize the phosphorylation site. We performed mass spectrometry analysis of immunoprecipitated LYL1-V5 digested with endoproteinase Lys-C and trypsin. Separately, using MALDI-TOF mass spectrometry, we measured the mass-to-charge (m/z) ratios of peptide fragments generated either from the upper or the lower band of LYL1. The sequence of each identified fragment was confirmed by MS/MS analysis. After comparing the m/z of corresponding peptides we found that the only difference between the phosphorylated and the non-phosphorylated forms of LYL1 is between the peptides spanning residues 34 through 48. The expected m/z of peptide 34–48 is 1556.8 which was the m/z measured for this fragment when derived from the lower band or non-phosphorylated LYL1 (Fig. 2B). When derived from the upper band or phosphorylated LYL1, the m/z of this fragment was 1636.8. In both cases we also observed traces of either the phosphorylated or the non-phosphorylated forms which was most likely due to cross contamination during the excision of the bands. The m/z difference of 80 between the upper and lower fragments suggests that the fragment from the phosphorylated LYL1 has a single phosphate group modification. The only place this modification could occur is at S_36_ (Fig. 2B). The phosphorylation at S_36_ was further confirmed by a single residue substitution. Expression studies showed that the upper band of LYL1 disappeared completely upon substitution of S_36_ with alanine (LYL1-S36A) (Fig. 2C).

After localizing the phosphorylation site we focused on identifying the kinase responsible for the phosphorylation of LYL1. Serine 36 with Proline residues at the +1 and the -2 positions (PASP) represents a classic mitogen-activated protein kinase (MAPK) phosphorylation site [26], [27]. Therefore, we tested if MAPK can phosphorylate LYL1 and if S_36_ is the target site. In order to do so, we immunoprecipitated wild type LYL1 (LYL1-WT) and LYL1-S36A from 293T cells, dephosphorylated them with Protein phosphatase 1 (PP1) and rephosphorylated with MAPK. The MAPK rephosphorylated successfully only LYL1-WT. There was no evidence suggesting phosphorylation of LYL1-S36A. Our results indicate not only that MAPK phosphorylates LYL1 but that the phosphorylation site is S_36_ (Fig 2D).

### The proteasome degrades LYL1 in a phosphorylation-independent manner

**Figure 3:**
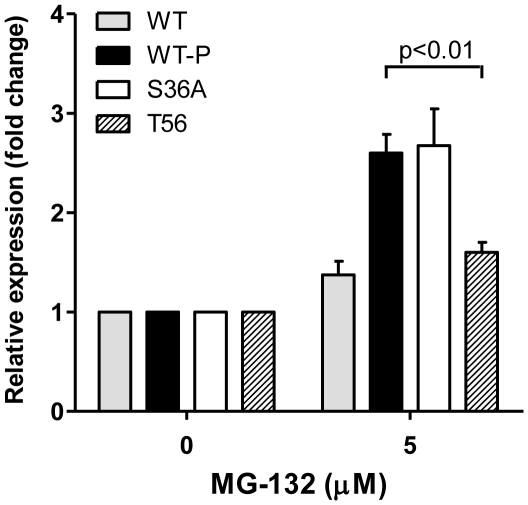
Effect of MG-132 inhibition of the proteasome on the expression of LYL1-WT and variants. Transiently transfected 293T cells were treated with DMSO as a control, or 5 µM MG-132 as previously described. LYL1 protein expression was analyzed as described in Fig. 1.

Protein phosphorylation is a well recognized mechanism for regulation of degradation by the proteasome [25], [28], [29]. Other bHLH transcription factors, such as c-Myc and Scl also exhibit phosphorylation-dependent proteasomal degradation [30], [31]. In order to assess if phosphorylation has a role in the proteasomal degradation of LYL1 we treated 293T cells, transiently transfected with LYL1-WT or LYL1-S36A with 5 µM MG-132 following the same procedures as before. The MG-132 induced accumulation of the non-phosphorylated (S36A) form of LYL1 was the same as the accumulation of LYL1-WT indicating that there is no difference in the degradation of LYL1 whether it is phosphorylated on not (Fig. 3). In addition, if phosphorylation was required for degradation, we should observe cellular accumulation of LYL1-S36A when transiently expressed in 293T cells (Fig. 2C). Some increase of the band intensity of the non-phosphorylated S36A form was expected because, it represents a combination of the upper and lower bands given by the LYL1-WT. It is apparent that the proteasome degrades LYL1 regardless of its phosphorylation state.

### The N-terminus of LYL1 contains a PEST rich degradation site

**Figure 4:**
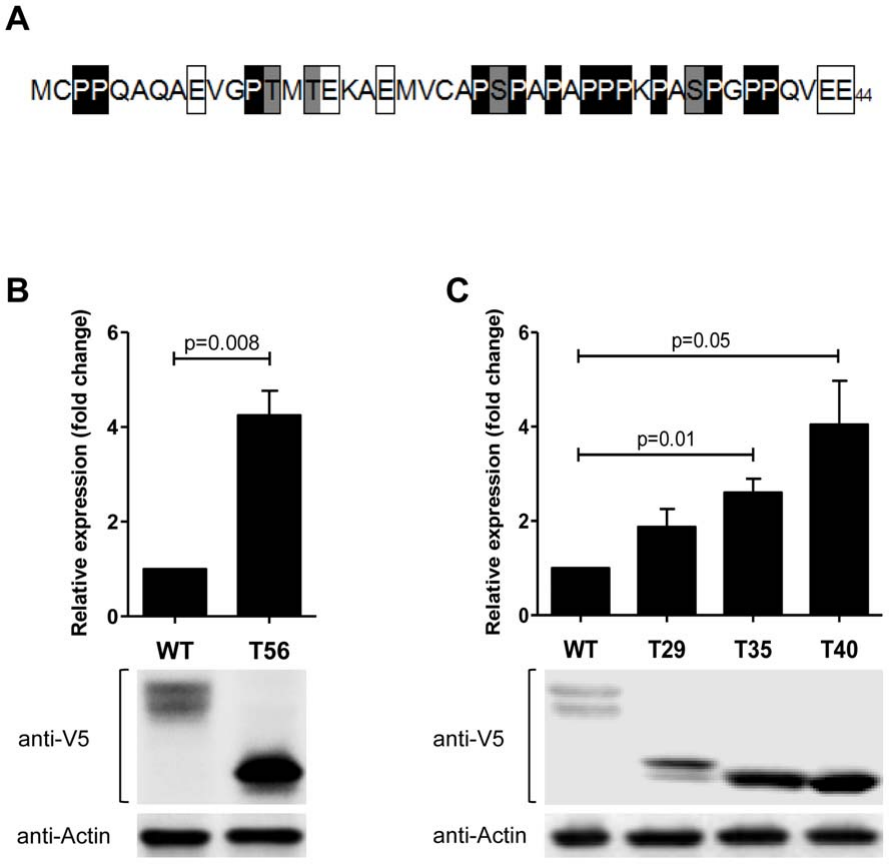
LYL1 degradation is dependent on a PEST motif located at the N-terminus of LYL1. **A**) Sequence of the first 44 amino acids of LYL1. The PEST residues have been highlighted or boxed for easier identification and convenience. **B**) Comparative expression in 293T cells of transiently expressed wild type (WT) and T56 or additional truncated forms of LYL1(**C**). The bar graphs describe the average band intensity for each protein from three or four experiments. The SEM is represented by the error bars. The immunoblotting for β-Actin serves as a control. The p value was calculated using two tailed, paired t test.

Since phosphorylation does not affect LYL1 degradation, we focused our attention on identifying degradation signals within the sequence of LYL1. The presence of a PEST rich sequence motif has been associated with a short protein half-life and a higher probability for degradation [20], [21]. Protein sequence analysis revealed that 22 or 50% of the first 44 residues of LYL1 are PEST residues (Fig. 4A): 13 Proline (29.5%); 5 Glutamate (11.4%); 2 Serine (4.5%) and 2 Threonine (4.5%) amino acids. To investigate the role of the PEST amino acid cluster we compared the expression of the wild type LYL1 to a truncated form of LYL1 lacking the PEST rich sequence. We observed a 4.3 fold increase in LYL1 expression upon deletion of the initial 56 amino acids (LYL1-T56) (Fig 4B). The accumulation was most likely due to inefficient degradation of LYL1 by the proteasome. This statement was further supported by our observation that there was no significant accumulation of LYL1-T56 as a result of treatment with 5 µM MG-132 (Fig. 3) suggesting that in the absence of the first 56 residues, LYL1 degradation becomes significantly less dependent on the proteasome. Clearly, the N-terminus of LYL1 contains a PEST rich sequence-motif responsible for the stability and degradation of LYL1.

After a closer examination of the 44 residue segment rich with PEST amino acids, we noticed that 10 of the 13 total Proline residues are located between amino acids 24 and 40. The Prolines account for 59% of all residues in that segment. The presence of such a high number of Prolines would destabilize any secondary structures and most likely be a part of an unstructured loop [32], which are often preferred targets for degradation [21], [33]. To achieve more precise localization of the degradation site and to investigate the role of the Proline cluster, we constructed additional truncated forms of LYL1. Deletion of the first 29 amino acids, or 3 of the 10 Proline residues in the 24–40 segment caused a noticeable but, not significant increase of LYL1 expression. After removing 6 additional residues (LYL1-T35) or 7 of the 10 Prolines, LYL1 expression increased significantly (2.6 fold) compared to the wild type (Fig 4C). The deletion of 5 more residues (LYL1-T40), or all 10 Prolines, resulted in over 4 fold increase of LYL1 expression which is almost identical to the expression of the T56 truncated form of LYL1 (Fig 4B). It appears that the cluster of Proline residues between amino acids 24 and 40 is instrumental in driving LYL1 degradation.

## Discussion

In this study we examined the mechanisms governing the protein stability and degradation of LYL1. We showed that LYL1 is degraded by the proteasome. Interestingly, we observed that only the upper band of the wild type LYL1 accumulated during a proteasomal block with MG-132. Additional studies revealed that the upper band of LYL1 represents the phosphorylated form of LYL1. Furthermore, we established that LYL1 is phosphorylated by the MAPK and identified S_36_ as the phosphorylation site. However, phosphorylation did not appear to be required for proper LYL1 degradation by the proteasome. The expression of the LYL1-S36A was just as dependent on the function of the proteasome as the LYL1-WT. The question of why the phosphorylated LYL1 accumulates and the non-phosphorylated LYL1 does not, or at least does not begin to show signs of accumulation until the use of higher concentration of MG-132, still remains unanswered. One possible explanation is that the phosphorylation does not have signaling role with respect to degradation but, simply under physiological conditions, the phosphorylated form of LYL1 might be the form that reaches the proteasome for degradation hence that is the form which predominantly accumulates.

**Figure 5:**
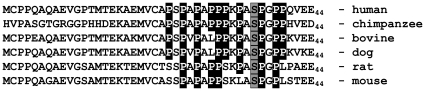
Alignment of N-terminal protein sequences of LYL1. The first 44 amino acids obtained from the human (NP_005574), chimpanzee (XP_524130), bovine (NM_001193074), dog (XP_853573), rat (NP_001007678) and mouse (NP_032561) protein sequences were aligned. All Proline residues from position 24 to 40 were highlighted as well as Serine 36.

While the degradation is phosphorylation-independent, the absence of the PEST motif significantly decreases the ability of the proteasome to degrade LYL1, leading to protein accumulation. More detailed studies revealed that a cluster of Proline residues located between amino acids 24 and 40 is essential for the proper degradation of LYL1. Clearly, the proteasomal degradation of LYL1 depends on a PEST and more specifically on a Proline rich sequence located in the N-terminus of LYL1. We found further supporting evidence in the alignment of N-terminal protein sequences of LYL1 from six species (human, chimpanzee, bovine, dog, rat and mouse) (Figure 5). We observed significant conservation of the Proline residues from residue 24 to 40. All Prolines, but one in the bovine and dog sequences, were preserved in the higher mammals. The rodent sequences have fewer Prolines; however, their even distribution may still preserve the role of this segment as a degradation signal. The Serine 36 residue is conserved in all sequences. The MAPK site is also conserved with the exception of the mouse. The fact that Serine 36 and the MAPK motif are conserved suggests that MAPK phosphorylation may have a significant physiological role in the function of LYL1; however, we have no evidence suggesting that it has a role in LYL1 degradation.

The lack of proper degradation leads to accumulation of LYL1 which might not only increase the activity of LYL1 but may also provide an environment supporting positive selection of cells with increased potential to become transformed. Disregulated degradation of oncogenes is a proven mechanism for induction of cancer formations [16], [28], [30], [34]. Considering the results presented in this study, added to the fact that LYL1 is a transcription factor, it is plausible that factors, such as mutations or improper regulation, preventing LYL1 degradation or inactivation by the proteasome may have significant role in cancer formation.

## Materials and Methods

### DNA constructs

All constructs were prepared by insertion of coding sequences into the pcDNA-DEST40 vector (Invitrogen) using Gateway recombination techniques. The stop codons were removed from the open reading frames to allow C-terminal fusion of the expressed proteins with the V5 tag provided by the vector. The *LYL1* wild type cDNA was purchased from Open Biosystems and was used as a template for subcloning and preparation of all *LYL1* constructs.

### Cell culture

Cultured 293T cells were maintained in DMEM/F-12 (50/50 mix) growth media with L-glutamine and 15 mM HEPES (Mediatech, Inc), supplemented with 10% fetal bovine serum (HyClone) at 37°C in a humidified CO_2_ incubator. The active growth of the cells was maintained by regular subculture. Cells beyond 20 passages were not use.

### Transient transfections

Cultured 293T cells were transfected with plasmid DNA using Lipofectamine 2000 reagent according to the manufacturer's protocol (Invitrogen). The cells were harvested for subsequent applications 24 or 48 hrs after transfection.

### Immunoprecipitation (IP) experiments

Transfected 293T cells were washed with phosphate-buffered saline (PBS) (Fisher) and solubilized in IP buffer (PBS, pH 7.4, 2% IGEPAL (Sigma), supplemented, prior to use, with Protease Inhibitor Cocktail for use with mammalian cell and tissue extracts (Sigma)). The lysates were passed 13 times through a 25 G needle and centrifuged at maximum speed for 8 min at 4°C in an Eppendorf microfuge. The clarified lysates were incubated with 2 µg of anti-V5 (Invitrogen) antibody for 30 min. followed by incubation for additional 60 min. with 25 µl of a 50% slurry of Protein A/G Plus agarose (Santa Cruz Biotechnology). After the incubations, the beads were washed three times with 400 µl of IP buffer. The precipitate was solubilized in SDS sample buffer (Bio-Rad) and resolved on 10% or 4–20% Tris-HCl Rgels (Bio-Rad). The gels were immunoblotted using anti-V5 or anti-β-Actin (Santa Cruz Biotechnology) monoclonal antibodies. Immunoblots were developed with the ECL Plus chemiluminescence reagent (Amersham). They were visualized with a Storm 860 phosphorimager, and the band intensities were quantified using Image Quant software (GE Healthcare).

### Treatment with MG-132

Transiently transfected 293T cells were incubated (24 hrs post-transfection) in culture media, supplemented with 2.5 or 5 µM MG-132 (Calbiochem), or DMSO as a control for 6 hrs. After the incubation, the cells were solubilized in IP buffer and equal amounts of lysate and 2X SDS sample buffer (Bio-Rad) were mixed followed by boiling for 10 min. The protein concentration of the non-denatured lysate was measured using the BCA Protein Assay Kit (Thermo). Equal amount of total protein was loaded on a 10% Tris-HCl Rgel for expressions analysis by Western immunoblotting.

### Dephosphorylation with Alkaline phosphatate

LYL1-WT was transiently expressed in 293T and then immunoprecipitated. Following the incubation with the Protein A/G beads, the samples were washed twice with IP buffer, once with PBS and once with NEBuffer 3 (New England Bio.). Each sample was then resuspended in 15 µl of NEBuffer 3 and 10 U of Calf Intestinal Phosphatase (CIP) (New England Bio.). Next, the samples were incubated at 37°C for 1 h with periodical mixing (every 10–15 min.) by flicking. Following the incubation, to the samples were added 20 µl of SDS-PAGE sample buffer (Bio-Rad) and they were boiled for 10 min. LYL1 was resolved by SDS-PAGE (10% Rgel, Bio-Rad), followed by immunoblotting.

### Mass Spectrometric Analyses

Immunoprecipitated LYL1-WT was resold by SDS-PAGE and the gel was stained for 60 min. using GelCode Blue Stain Reagent (Thermo). After 60 min. distaining in distilled water the phosphorylated and non-phosphorylated bands were excised and submitted for mass spectrometric analysis to the Proteomics Core at Baylor College of Medicine. The gel bands were then rinsed in H_2_O for 10 min, cut with a scalpel blade into ∼1 mm pieces, dehydrated with 0.2 M TRIS pH 8 containing 50% acetonitrile for 30 min and dried completely in a Speed-Vac. Next, the gel pieces were rehydrated in 0.05 M TRIS pH 8 containing 0.5–1 µg each modified trypsin (Promega) and Lys-C (Wako) and digested for 20 hr at 37°C. The supernatants were removed to a clean microfuge tube, the gel fragments were extracted with aqueous 50% acetonitrile/1% formic acid for ∼15 min and the extract combined with the supernatant. Samples were then evaporated to ∼10 µl, acidified with formic acid to ∼pH 3 and desalted on a C_18_ ZipTip (Millipore). Peptides were eluted from the ZipTip with 3–5 µl of an aqueous solution of 50% acetonitrile containing 2% formic acid and spotted on a MALDI target plate with matrix (HCCA, alpha-cyano-4-hydroxycinnamic acid), dried and analysis performed in reflector mode on an ABI/SCIEX 4700 Proteomics Analyzer TOF/TOF mass spectrometer. Monoisotopic peptide masses detected were analyzed by MS-Fit (Protein Prospector, University of California, San Francisco) for protein database searches and protein identification/verification. Spectra were visually inspected for the presence of peptide mass differences between the 2 samples. The selected peptide precursor ions were subjected to high-energy collision induced dissociation to generate MS/MS fragment ion spectra that were analyzed and confirmed by visual inspection to deduce amino acid sequences of the peptides.

### MAPK phosphorylation

LYL1-WT and LYL1-S36A were immunoprecipitated from transiently transfected 293T lysates as described above. Following the incubation with the beads, the samples were washed twice with IP buffer, once with PBS and once with NEBuffer for Protein MetalloPhosphatases (PMP) supplemented with MnCl_2_. Next, the samples were resuspended in 15 µl of NEBuffer PMP supplemented with MnCl_2_ and to the two of the three samples in each set were added 5 U of Protein Phosphatase 1 (PP1) (New England Bio.). All samples were incubated at 30°C for 2 hrs with periodical mixing. After the incubation all samples were washed once with IP buffer, once with PBS and once with Kinase Buffer (NEBuffer for Protein Kinases (PK) supplemented with 1 mM ATP and 1 µM Protein phosphatase inhibitor 2). After the wash, the samples were resuspended in 15 µl of Kinase Buffer and to one of the two dephosphorylated samples in each set were added 100 U of MAPK. The samples were then incubated for 1–2 hrs at 30°C. Following the incubation the samples were washed once with IP buffer and once with PBS. After the final wash the precipitated proteins were released from the beads by addition of SDS-PAGE sample buffer (Bio-Rad) followed by boiling for 10 min. The proteins were resolved by SDS-PAGE, followed by immunoblotting.
